# Testing a Method Based on an Improved UNet and Skeleton Thinning Algorithm to Obtain Branch Phenotypes of Tall and Valuable Trees Using *Abies beshanzuensis* as the Research Sample

**DOI:** 10.3390/plants12132444

**Published:** 2023-06-25

**Authors:** Jiahui Shen, Lihong Zhang, Laibang Yang, Hao Xu, Sheng Chen, Jingyong Ji, Siqi Huang, Hao Liang, Chen Dong, Xiongwei Lou

**Affiliations:** 1 College of Mathematics and Computer Science, Zhejiang A & F University, Hangzhou 311300, China; 2021611011046@stu.zafu.edu.cn (J.S.); lhao@zafu.edu.cn (H.L.); 2 Key Laboratory of State Forestry and Grassland Administration on Forestry Sensing Technology and Intelligent Equipment, Zhejiang A & F University, Hangzhou 311300, China; 3 Key Laboratory of Forestry Intelligent Monitoring and Information Technology Research of Zhejiang Province, Zhejiang A & F University, Hangzhou 311300, China; 4 Longquan Forestry Bureau, Longquan 323700, China; 202005100122@stu.zafu.edu.cn (L.Z.); 20010036@zafu.edu.cn (J.J.); 5 Hangzhou Ganzhi Technology Co., Ltd., Lin’an 311300, China; wgsdgh@zafu.edu.cn; 6 Zhejiang Forestry Bureau, Hangzhou 310000, China; xuh.lyt@zj.gov.cn; 7 Center for Forest Resource Monitoring of Zhejiang Province, Hangzhou 310000, China; chens.lyt@zj.gov.cn; 8 Longquan Urban Forestry Workstation, Longquan 323700, China; meilihuang@zafu.edu.cn

**Keywords:** phenotype monitoring, tall tree, improved UNet model, skeleton algorithm, branch measurement

## Abstract

Sudden changes in the morphological characteristics of trees are closely related to plant health, and automated phenotypic measurements can help improve the efficiency of plant health monitoring, and thus aid in the conservation of old and valuable tress. The irregular distribution of branches and the influence of the natural environment make it very difficult to monitor the status of branches in the field. In order to solve the problem of branch phenotype monitoring of tall and valuable plants in the field environment, this paper proposes an improved UNet model to achieve accurate extraction of trunk and branches. This paper also proposes an algorithm that can measure the branch length and inclination angle by using the main trunk and branches separated in the previous stage, finding the skeleton line of a single branch via digital image morphological processing and the Zhang–Suen thinning algorithm, obtaining the number of pixel points as the branch length, and then using Euclidean distance to fit a straight line to calculate the inclination angle of each branch. These were carried out in order to monitor the change in branch length and inclination angle and to determine whether plant branch breakage or external stress events had occurred. We evaluated the method on video images of *Abies beshanzuensis*, and the experimental results showed that the present algorithm has more excellent performance at 94.30% MIoU as compared with other target segmentation algorithms. The coefficient of determination (R^2^) is higher than 0.89 for the calculation of the branch length and inclination angle. In summary, the algorithm proposed in this paper can effectively segment the branches of tall plants and measure their length and inclination angle in a field environment, thus providing an effective method to monitor the health of valuable plants.

## 1. Introduction

Plant phenotype refers to all observable characteristics of plants that allow for the measurement of plants’ various structural and functional characteristics [1]. For research on precious trees, whose spatial distribution determines the morphology of trees, the fast and accurate extraction of tree morphological information plays an essential role in determining their health status. The *Abies beshanzuensis* is an ancient relic plant endemic to China, with only three surviving native individuals located in the Zhejiang Province, China [2]. It is a typical species facing the mountain-top extinction crisis [3], listed by the International Union for Conservation of Nature Species Survival Commission (IUCN-SSC) in 1987 as one of the 12 most endangered plants in the world. With global climate change, *Abies beshanzuensis* is also facing a decrease in available habitat and an increased risk of extinction [4]. Therefore, it seems necessary to monitor its health status in real time by analyzing its morphological changes for phenotypic parameters. As the main structural components of plants, the trunk and branches play a crucial role in supporting the plant and in regulating its growth and development. Many parameters describing the trunk and branches, such as diameter at breast height, length, and inclination, are widely used as indicators for measuring the health status of plants [5]. Changes in tree branch morphology can have a critical impact on its growth and health. The length and inclination of branches can affect the efficiency of photosynthesis and nutrient uptake, and they may also lead to the breakage and torsion of the tree itself [6]. In recent years, the advancements in digital imaging technology and deep learning has allowed these to be widely used in medicine, remote sensing, and intelligent agriculture [7,8,9]. Therefore, this is a promising approach for non-destructive plant phenotype monitoring that is based on images and computer vision as it can automatically record traits and reduce the manual workload in complex environments in the field [10].

Obtaining information on a plant’s branches is a prerequisite for monitoring its health. In early works on detecting tree trunks and branches, many scholars used images and point clouds. Nagham et al. [11] used cameras and laser scanners to detect the branches of fruit trees, achieving a detection accuracy of 96.64%. However, the study examined the trunk of the tree but did not take into account the branching. Ji et al. [12] used a contrast limited adaptive histogram equalization to identify the branches. The identification rate reached 94%, better than OTSU (maximum between-class variance) and the histogram algorithm. Amatya et al. [13] utilized a Bayesian classifier to segment branching pixels in images, grouped the branches in specified neighborhoods after filtering out the noise, and connected them using a curve-fitting method. The overall accuracy was 89.2% for individual branches. Zhang et al. [14] introduced a method for extracting topological and structural information from fruit tree branches based on LIDAR (light detection and ranging) point clouds. They also applied the QSM method to study fruit tree structure and realized the hierarchical automatic extraction of fruit tree branches and trunks. However, due to the complexity of the actual field environment, traditional computer vision-based detection methods are affected by light and noise, while point-cloud-based identification methods are heavily equipment-dependent, with laser sensors being expensive and performing poorly in real time. These have limited their applications in monitoring plant phenotypes.

With the development of artificial intelligence, object detection and segmentation technology based on deep learning has been gradually applied to tree branch identification and has achieved good accuracy. Buzzy et al. [15] used the target detection algorithm, Tiny-YOLOv3, to detect, count, and localize plant leaves with an F1-score of more than 0.94. Tong et al. [16] used four deep learning algorithms to detect and segment the trunk, branches, and supports of apple trees, to extract the apple tree skeleton, and then to locate the nodes based on the results. Their results showed that when IoU is 0.5, the bbox mAP and segm mAP of Cascade Mask R-CNN Swin-T are the highest at 0.943 and 0.940, respectively. Yang et al. [17] proposed a segmental labeling method for random and irregular branches, using Mask R-CNN to train and segment differently labeled citrus branches. The accuracy of the segmental labeling reached 96.2%, which was 26.6% higher than the accuracy of the overall labeling method. Liang et al. [18] used the momentum-optimized stochastic gradient descent method as an optimizer based on UNet to segment Litchi’s fruits and stems, effectively improving the MIoU to 80.65%. Qiao et al. [19] proposed to embed the convolutional block attention module (CBAM) into MobilenetV2 as the backbone of PSPNet and then introduced the subdivision of the refinement residual Blocks (RRBs) into the main branch and side branch of the model to segment the red jujubes trunk. Compared with the original PSPNet, the intersection over union (IoU) value of the improved model increased by 0.67%. Lin et al. [20] proposed a real-time tree segmentation method with a branch IoU of 63.33% via an improved FCN. In the above study, the deep learning algorithm was successfully applied to various plant detection and segmentation applications, effectively addressing the limitations of the traditional machine vision methods.

Algorithms, such as CNN and FCN, have been used in existing studies to detect and segment trunks and branches, and good results have been achieved. Due to the complexity of deep learning models, limitations in computational power, algorithm optimization, and information overload have arisen. Therefore, we need to improve and optimize the network architecture to address these issues. UNet [21] was initially applied in medical image segmentation as an early CNN because UNet requires only a small amount of data to produce accurate segmentation results. UNet has also been applied in plant segmentation tasks by several researchers, who have proposed many enhancements to the UNet network structures to improve the semantic segmentation performance [22,23]. In addition, attention mechanisms have shown outstanding performance in the natural language processing (NLP) and computer vision tasks of the commonly used models. The attention mechanism can highlight expressive features and suppress the irrelevant ones [24,25], which significantly improves the accuracy of target detection and semantic segmentation, and its application has been well studied in different fields [26,27,28,29].

Currently, plant phenotypic studies are mainly concentrated on smaller plants, including crops, with fewer studies acquiring phenotypic information, such as the branch length and inclination angle of taller plants in natural environments with complex backgrounds and harsh weather. This study thus aimed to acquire plant branch phenotypic data in complex natural environments by utilizing semantic segmentation techniques and skeleton thinning algorithms. The research objectives were as follows:

(1) Based on the squeeze-and-excitation (SE) module [30] and residual attention module (RAM) [31], an improved UNet is proposed to construct a segmentation model for large plants.
(2) According to the segmentation results obtained using the improved model, image processing techniques and skeleton thinning algorithms are used to obtain the skeleton lines of the trunks and branches of the plant, and their lengths are calculated one by one.
(3) Based on the skeleton line, the inclination angles of the trunks and branches of the plant are obtained by fitting its straight lines using the Euclidean distance.

In this paper, the above model and algorithm were experimentally validated using video images of *Abies beshanzuensis*. The data on branch length and inclination angle in different seasons and weather were obtained and evaluated.

## 2. Materials and Methods

### 2.1. Experimental Site and Image Data Acquisition

**Figure 1:**
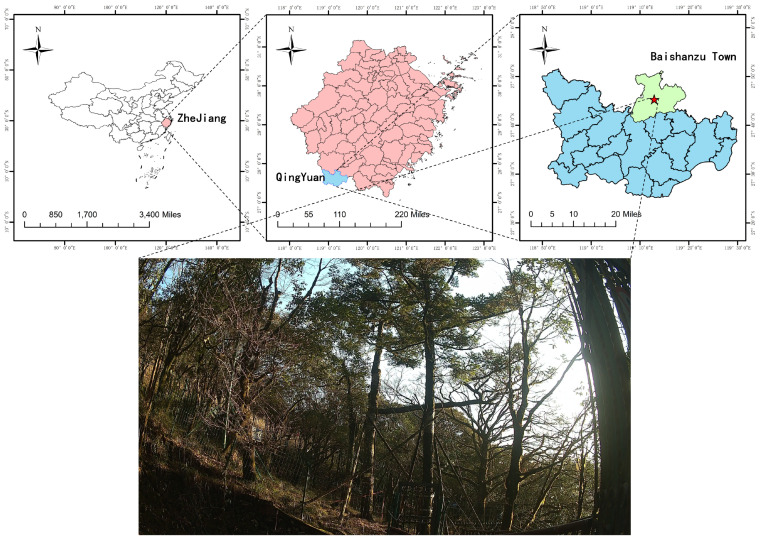
Abies beshanzuensis at the experimental site. The red star represents the location of the Abies beshanzuensis in Baishanzu Town.

The image data of *Abies beshanzuensis* used in the experiment were obtained from the core reserve of the Baishanzu National Park in Baishanzu Town, Qingyuan County, Lishui City, Zhejiang Province, China (27°4′–27°5′ N, 119°1′–119°2′ E; 1750 m), which has a typical subtropical monsoon climate. The experimental site is shown in Figure 1, where anthropogenic activities are prohibited to ensure the authenticity of the ecosystem. The average age of the natural *Abies beshanzuensis* is about 190 years, with a tree height of approximately 12 m and a breast diameter of around 29 cm. Since the diameters of some native branches were relatively small and there were a large number of other branches in the shooting background, two high-resolution color cameras (HIKVISION DS-2SK8C244IJY-LJJ) were used in this study to continuously acquire image information of the *Abies beshanzuensis* branches, with a maximum resolution of 2560 × 1440, in order to ensure the comprehensiveness and completeness of the samples. The cameras used two lenses, panoramic and detailed, which could take pictures of the *Abies beshanzuensis* from different angles, including during the daytime and nighttime and with good natural light. The sampling weather conditions were sunny, rainy, snowy, foggy, and typhoon. The image data were collected from July 2022 to January 2023. For the construction of the branch segmentation model, 400 image samples from July 2022 to January 2023 were selected for training. The training and test samples were divided at a ratio of 9:1. The training samples were labeled as trunk and branch using LabelMe, and the *Abies beshanzuensis* images were trained using the format of the VOC dataset. For the branch length and inclination measurements, a total of 2760 branch samples were selected for the experiment in various environments from August 2022 to January 2023.

### 2.2. Improvement of UNet Segmentation Model

#### 2.2.1. Original UNet Model

**Figure 2:**
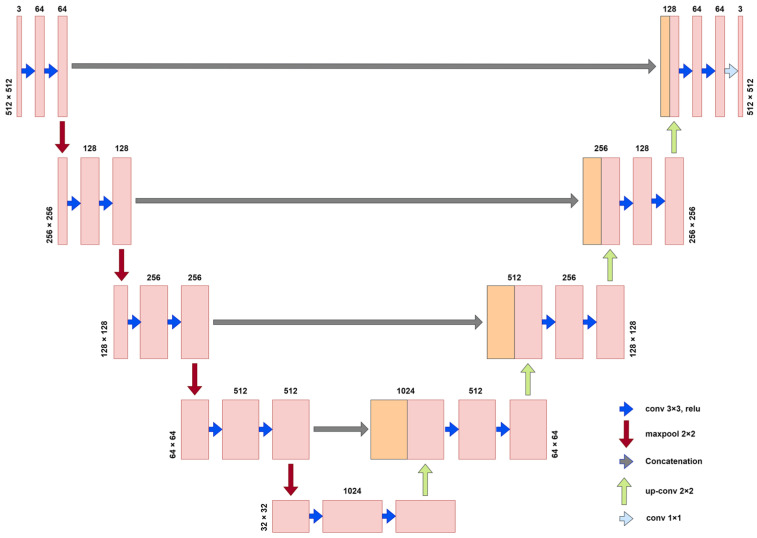
UNet network structure.

UNet was proposed by Ronneberger et al. at the 2015 MICCAI conference, and it utilizes a symmetric structure to enable the fusion of feature mappings between different levels. UNet can use a smaller number of training images to predict the class of pixel points and form more complex judgments. The UNet network structure diagram is shown in Figure 2. UNet consists of three parts: encoder (downsampling), decoder (upsampling), and skip connection. The encoder stage consists of two 3 × 3 convolutional layers and a 2 × 2 max-pooling layer with a stride of 2. The activation function utilized is the rectified linear unit (ReLU) function, which performs four downsampling operations. After each pooling operation, the size of the feature map is reduced to half of the original size, and the number of channels is doubled. The decoder stage and the encoder parts correspond to four upsample iterations through the 2 × 2 deconvolution layer (a transpose matrix). Each time the size of the upsampled feature map is doubled, the number of channels is halved. UNet fuses the features obtained from the encoder with those obtained in the decoder by skip connection and combines the features for refinement. The final output layer contains a 1 × 1 convolutional layer, followed by a Sigmoid activation function for pixel-level classification. Unlike other semantic segmentation networks, this network introduces a skip connection to fuse the adequate feature layers extracted by the backbone network with the height and width of the final output image, such that the height and width of the final output image are equal to those of the input image. Meanwhile, the apparent features of the generalized underlying features and the high-level features can be fused, which alleviates the vanishing gradient problem during the training process and simplifies the model to some extent. The encoder’s shallow structure can extract the topography’s simple features, while the deep structure can capture the complex features of the topography to retain more detailed information, making the segmentation results more refined and more easily adaptable to the complex information of the *Abies beshanzuensis* images in the field environment.

#### 2.2.2. Backbone Network Based on VGG16

To accelerate the convergence speed of the model, this study used the VGG16 model as the backbone feature extraction network for the UNet network. VGG16 consists of 5 convolution blocks and some fully connected layers; the first two convolution blocks have two convolution layers each, and the other three convolution blocks have three convolution layers each. When used, the max-pooling layer in the fifth convolution and the subsequent fully connected layers were removed from this paper. Therefore, this section consists of 13 convolutional layers and 4 max-pooling layers, with a kernel size of 3 × 3, a stride of 1, and zero-padding pixels. These convolutional layers automatically extract features from the images. After each convolutional layer, ReLU is used as the activation function. Pooling operations for the max-pooling layer were carried out using a kernel of size 2 × 2, with a stride of 2 and no padding. Compared with the original UNet model, three convolution depth layers were added so that the model could better extract the feature information from the trunk and branches of *Abies beshanzuensis*.

#### 2.2.3. Squeeze-And-Excitation Module

**Figure 3:**
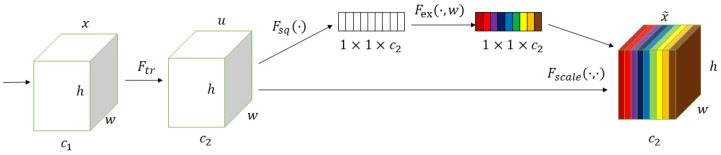
SE module.

Since the main trunk and branch parts of *Abies beshanzuensis* occupy a small proportion of the image, considerable computational resources are wasted in non-branch areas when using regular convolution. Therefore, we added the SE module to the encoder. The structure of the SE module is shown in Figure 3. The SE module contains two parts: a squeeze part and an excitation part. The squeeze operation performs feature compression along the spatial dimension using global average pooling and converts each two-dimensional feature channel to a real number. The excitation operation consists of two fully connected layers, a ReLU layer and a Sigmoid operation, which consider the dependency between the feature maps and recalibrate the importance along channels. The SE module can adaptively recalibrate channel-wise feature responses by explicitly modeling interdependencies between channels, and it automatically acquires the importance of each feature channel through model learning, and then strengthens useful features based on the importance of each channel while suppressing features that are not useful for the current task. By improving the quality of spatial encoding in the feature hierarchy of the network, the representational power of the network can be enhanced. Embedding the SE module into the downsampling part of the UNet can generally increase the data-fitting ability and improve the overall segmentation accuracy of the network.

In Figure 3, u is the input feature map, and h, w, $c_{2}$ are the height, width and number of channels, respectively. Firstly, the mth dimensional feature map $u_{m}$ is compressed (noted as $F_{sq}$), and the output is denoted as $z_{m}$. The calculation n equation is as follows:

(1) $$z_{m}=F_{sq}\left(u_{m}\right)=\frac{1}{wh}\sum_{i=1}^{w}\sum_{j=1}^{h}u_{m}\left(i,j\right)$$

i, j in Equation (1) are the coordinates of the feature map in the spatial dimension

The output of all feature maps after the squeeze operation is denoted as z. z is subjected to the excitation operation (denoted as $F_{ex}$), and the result is s. The calculation formula is as follows:

(2) $$s=F_{ex}\left(z,w\right)=\sigma \left(w_{2},\delta \left(w_{1},z\right)\right)$$

In Equation (2), w includes $w_{1}$ and $w_{2}$ as the weights of the fully connected layers, σ is the ReLU activation function, and δ is the Sigmoid activation function.

By multiplying the generated feature vector s with the corresponding channel of the feature map u, the operation is denoted as $F_{scale}$ and the output is $\tilde{x}$. The formula is as follows:

(3) $$\tilde{x}=F_{scale}\left(u_{m},s_{m}\right)=s_{m}u_{m}$$

#### 2.2.4. Residual Attention Module

This study examined the main trunk and branches of *Abies beshanzuensis*, which have a high similarity with the surrounding environment. In order to extract more features of *Abies beshanzuensis* in a complex environment, we introduced the residual attention module (RAM) before the first downsampling of UNet. The RAM is a structure that puts channel attention (CA) and spatial attention (SA) in parallel and combines them to increase the perception of features with higher contribution and value, thus improving the recognition ability of the model.

After the input feature map was convolved, and ReLU activated, the feature map was fed into the CA and SA units. In the CA unit, variance pooling was used, which allows for better attention to detail information as compared to the commonly used average pooling. In the SA unit, the attention map could be obtained by convolving the features for each channel separately, using a deep convolution with a kernel size of 3 × 3. The attention maps of CA and SA were summed to obtain the fused attention (FA) map, normalized to between 0 and 1 using Sigmoid, multiplied with the feature maps after two convolutions, and then summed with the input feature maps to finally generate the output.

**Figure 4:**
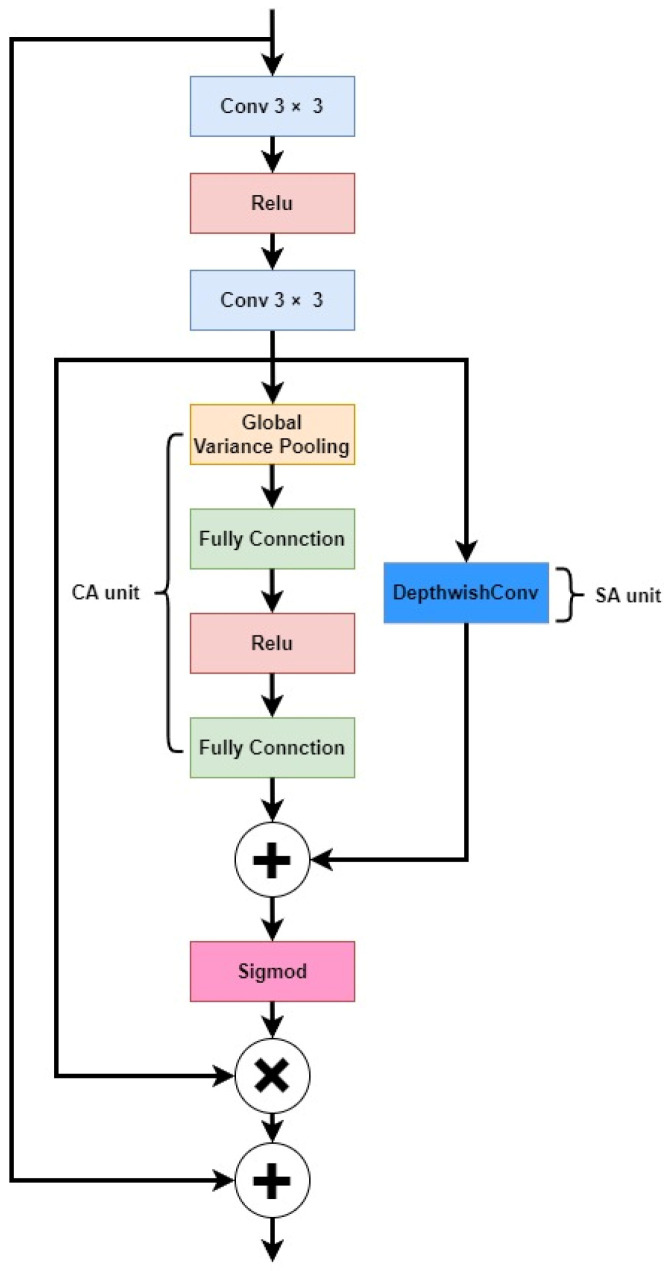
Residual attention module.

RAM is characterized by CA utilizing variance pooling and SA utilizing deep convolution, which is better suited for low-level tasks that require a focus on intricate details. This characteristic of RAM is essential for small-sample learning situations. The network structure of RAM is shown in Figure 4.

#### 2.2.5. Improved UNet Model

**Figure 5:**
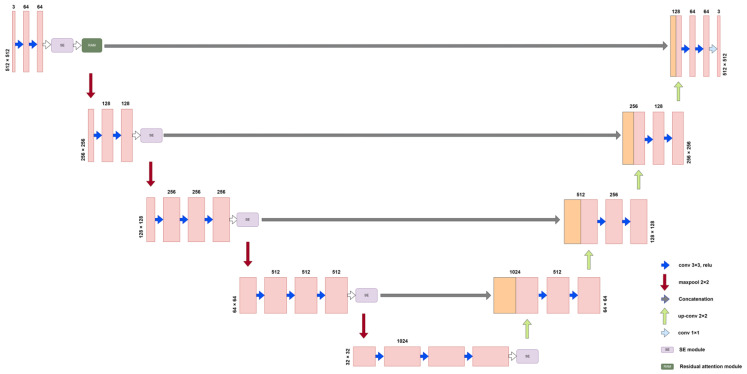
Improved UNet network structure.

Our improved model retains the original structure of the UNet end-to-end, which also consists of an encoder, decoder, and a skip connection. The model uses VGG16 as the encoder, increases the network depth, and consists of 4 sets of downsampling layers with convolutional layers, all of which have a convolutional layer size of 3 × 3. The decoder consists of four upsampling layers and a convolutional layer with the same parameters as the coding module and uses Softmax as the classification layer in the last layer to segment the image into background, trunk, and branch using a 1 × 1 convolutional output. There are skip connections between the encoder and decoder at each one of the four layers. The model introduces the SE module before each downsampling layer and the first five upsampling layers. It automatically obtains the importance of each feature channel by model learning, reducing the channels with weak feature expression, and strengthening the channels with strong feature expression. RAM is introduced after the first SE module is added to the model to obtain more detailed feature information. Figure 5 shows the improved UNet network structure.

### 2.3. Branch Length and Inclination Measurement Based on Zhang–Suen Thinning Algorithm

After the separation of the branches, the morphology of the branches can be monitored by the magnitude of the morphological changes to determine whether the plant is in a normal state. The morphological changes of the branches are mainly reflected by the changes in their length and angle, so the branches primarily need to be skeletonized. First, the image needs to be preprocessed to obtain a binary image of the branches of *Abies beshanzuensis*. Then, the skeleton structure of *Abies beshanzuensis* is extracted using the skeleton extraction algorithm. Cuevas-Velasquez et al. [32] evaluated five algorithms to extract the rose meristem skeleton. Among them is Zhang–Suen’s [33] method, which creates fewer burrs in the skeletonizing process, runs faster, and achieves an F1-score of 91.06%. Therefore, the Zhang–Suen algorithm was chosen to extract the branch skeleton in this study. 

**Figure 6:**
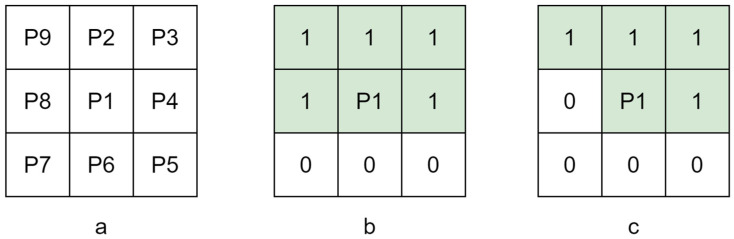
Pixel diagram: (**a**) 3 × 3 pixel window; (**b**) the pixel window that satisfies the first traversal; (**c**) the pixel window that satisfies the second traversal.

The Zhang–Suen thinning algorithm is an iterative algorithm that creates a 3~×~3 window and finds target pixels that meet specific conditions by traversing the central pixel, P1, and its adjacent pixels and then deleting them, as shown in Figure 6. The whole iterative process of the algorithm is divided into two steps. Step 1: The points that satisfy Equations (4)–(6) are deleted. Step 2: The points that satisfy Equations (4), (5) and (7) are deleted. The above two steps are continuously cycled until the target after the last deletion in the current round of operations is identified, no new pixel points are deleted, and the algorithm ends; the output is the skeleton of the binary image after refinement.

(4) $$2\leq N\left(P1\right)\leq 6$$

(5) $$S\left(P1\right)=1$$

(6) P2×4×P6=0, P4×P6×P8=0

(7) P2×P4×P8=0, P2×P6×P8=0

Here, N(P1) indicates the number of pixels with a value of 1 among the 8 pixels adjacent to P1; S(P1) indicates the cumulative number of occurrences of 0 to 1 from pixels P2 to P9 to P2.

In this study, the branch masks in the images were obtained based on the prediction results of the improved UNet model. Subsequently, the trunk and branches were distinguished by color threshold segmentation, and then the individual targets (trunk and each branch) were identified through a series of image processing steps. Finally, the individual target skeletons were extracted using the Zhang–Suen algorithm. The length of the target was obtained by calculating the number of pixel points in the image for the generated skeleton line. Calculating the angle requires fitting the skeleton to a straight line, and we chose the common Euclidean distance (Equation (8)) for the linear fit, which is easy to calculate and which captures the variation in the skeleton line well. The points on the skeleton line were then used as input. The skeleton line was fitted using the Euclidean distance to minimize the sum of the distances from the input points to the fitted line, to obtain the slope of the line and to calculate the inclination of the target using a trigonometric relationship.

(8) $$d=\sqrt{{\left(x_{2}-x_{1}\right)}^{2}+{\left(y_{2}-y_{1}\right)}^{2}}$$

### 2.4. Evaluation Indicators

In order to evaluate the branch segmentation in the *Abies beshanzuensis* images, four evaluation metrics were used here: mean intersection over union (MIoU), precision, recall, and F1-score. The formulas of these evaluation metrics are as follows:

(9) $$MIoU=\frac{1}{K+1}\sum_{i=0}^{k}\frac{TP}{FN+FP+TP}$$

(10) $$Precision=\frac{TP}{TP+FP}$$

(11) $$Recall=\frac{TP}{TP+FN}$$

(12) $$F_{1}-score=2*\frac{precision*recall}{precision+recall}$$

In addition, two metrics were used to quantify the estimation performance of the branch length and the inclination angle of *Abies beshanzuensis*: the coefficient of determination (R^2^) and the root mean squared error (RMSE), respectively. The equations are as follows:

(13) $$R^{2}=1-\frac{\sum_{i=1}^{n}{\left(t_{i}-m_{i}\right)}^{2}}{\sum_{i=1}^{n}{\left(t_{i}-{\overset{-}{t}}_{i}\right)}^{2}}$$

(14) $$RMSE=\sqrt{\frac{1}{n}\sum_{i=1}^{n}{\left(t_{i}-m_{i}\right)}^{2}}$$

Here, K is the number of categories, TP is true positive, TN is true negative, FP is false positive, FN is false negative, n is the number of test images, $t_{i}$ is the tilt of the manually measured *Abies beshanzuensis* branches in image i, $m_{i}$ is the tilt estimated by the method in this paper, and ${\overset{-}{t}}_{i}$ is the average of $t_{i}$.

## 3. Results

### 3.1. Experimental Platform and Model Training

In this study, the neural network training was fine-tuned based on pre-training weights, and “Adma” was selected as the optimizer for the training. The loss function was “CrossEntropy Loss”. The initial learning rate was set to 0.0001, and the learning rate was decreased by “cos”. After several training and debugging sessions, the batch size was set to 4, and epochs were set to 300, depending on the performance of the graphics card.

This study was coded in Python3 and tested using the TensorFlow2 deep learning framework with the following hardware and software configuration of the computers for model training and testing: the CPU was Intel Core i7-10700 F, the GPU was 12 G Nvidia GTX3060, the operating system was WIN10, and the parallel computing architecture was CUDA11.0.

### 3.2. Different Model Segmentation Results

**Figure 7:**
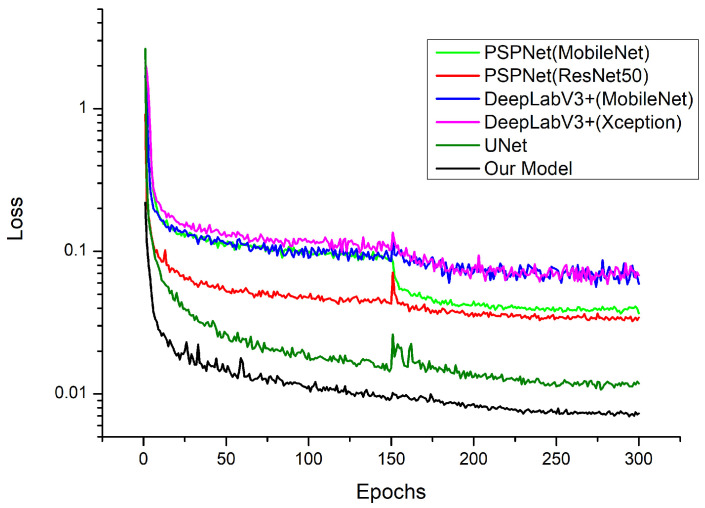
Variation curves for the training set loss values of six algorithms with epochs.

The model in this paper replaced the UNet backbone and added SE modules and RAM. To verify the validity of the model, it was compared with the original UNet and two semantic segmentation models paired with different backbones under the same testbed conditions; these were PSPNet (MobileNet, ResNet50) and DeepLabV3+ (MobileNet, Xception). The variation curves of the loss function for each network model are shown in Figure 7. The training was divided into two stages, namely the freezing stage and the unfreezing stage. The first 150 epochs froze the training parameters, and the feature extraction network did not change. A small amount of video memory was occupied, and only network fine-tuning was performed. The last 150 epochs unfroze the training parameters. At this time, the backbone of the model was not frozen, the memory occupied was significant, and all the parameters of the network were changed. As seen in Figure 7, a good training process can be achieved for each model.

**Figure 8:**
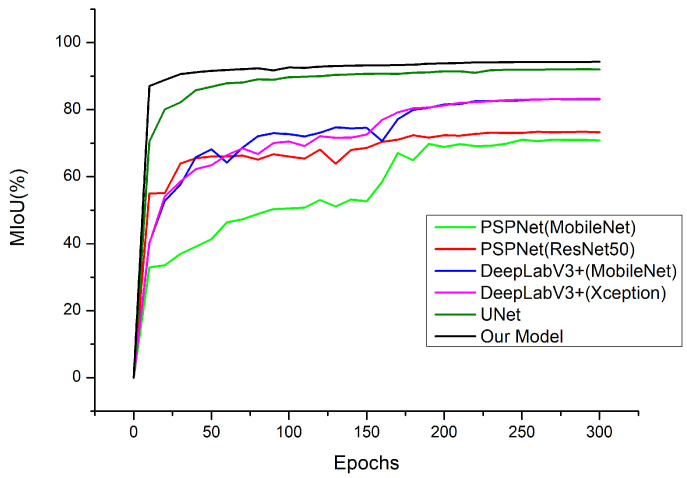
Variation curves of MIoU with the number of iterations for six models.

The variation curves of MIoU with epoch for the six models are shown in Figure 8. With the increase in epochs, the MIoU of each algorithm gradually converges. The MIoUs of the six models differed greatly at the initial stage. The improved UNet model and the original UNet model had higher MIoU at the early stage of the training and gradually stabilized after 20 epochs. Throughout the process, the improved UNet model converged faster than the other networks. It rose rapidly at the early stage of the MIoU training, improved slightly in the mid-term, and tended to be stable at the later stage, reflecting the rapid convergence and stability of the model.

**Figure 9:**
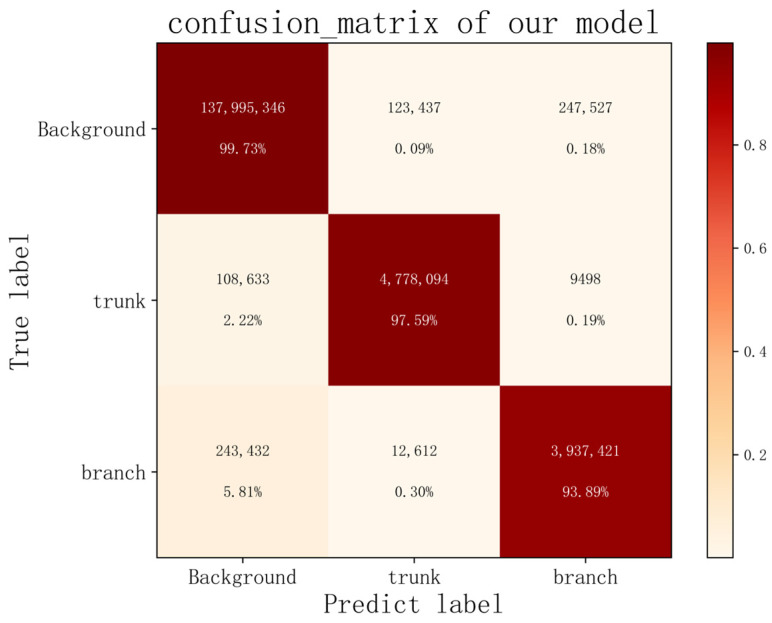
The confusion matrix of our improved model.

Table 1 gives the four metrics of the six models. Table shows that the improved branch and trunk segmentation network in this paper has the best segmentation performance. The MIoU of the segmentation of the branches of *Abies beshanzuensis* reached 94.2998%. The original UNet model outperformed the PSPNet and DeepLabV3+ models in all metrics. This is because the structure of *Abies beshanzuensis* is relatively fixed, and the semantic information is simpler, so both high-level semantic information and low-level features in this image are essential. UNet uses a skip connection to fuse the low-dimensional semantic features extracted at the encoding stage with the high-dimensional semantic features in order to obtain multi-scale features and achieve better segmentation results. Among them, the evaluation metrics obtained via PSPNet are relatively low, which may be due to the use of the global pyramid pooling module [34]. Although it increases the receptive field by using different pooling sizes, some of the branches have small areas, which may cause it to miss some feature information and lead to poor detail segmentation aspects, which then affects the performance of PSPNet. In DeepLabV3+, the inclusion of atrous convolution [35] in the network likewise leads to an increase in the receptive field of the convolution layer, which affects the performance. Compared with PSPNet and DeepLabV3+, UNet uses a more concise network structure and achieves better results. Compared with the original UNet model, the improved algorithm in this paper improved the MIoU by 2.2403%, precision by 1.1447%, recall by 1.359%, and F1-score by 1.2521%. The confusion matrix of the improved model is shown in Figure 9.

**Figure 10:**
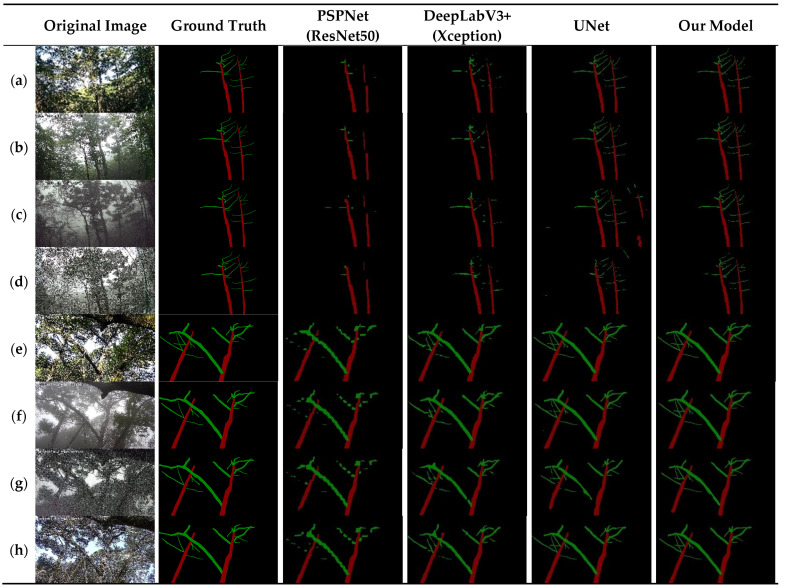
Segmentation results for *Abies beshanzuensis* trunk and branches. (**a**) Sunny day image from the first camera. (**b**) Rainy day image from the first camera. (**c**) Good natural light image from the first camera at night. (**d**) Snow day image from the first camera. (**e**) Sunny day image from the second camera. (**f**) Rainy day image from the second camera. (**g**) Good natural light image from the second camera at night. (**h**) Snow day image from the second camera.

In order to verify the effectiveness of this paper’s method for the semantic segmentation of *Abies beshanzuensis* branches, the results of the semantic segmentation were tested on the same set of images using the four models. Among them, ResNet50 was selected as the backbone network for PSPNet, and Xception was selected as the backbone network for DeepLabV3+. The segmentation effect is shown in Figure 10.

The figure shows that PSPNet and DeepLabV3+ have poor segmentation ability, and many branches cannot be segmented. It can be observed that PSPNet could hardly segment the branches effectively, and the branch part is obviously jagged. The segmentation of the trunk part is also obviously broken, which is not in line with reality. DeepLabV3+ can segment more targets than PSPNet, and the segmentation result map shows fragmented prediction parts in more places. There are also trunk and branch breaks, and the segmentation results are less satisfactory. DeepLabV3+ can segment more targets compared to PSPNet, and the segmentation result map shows fragmented prediction parts in more places. There are also trunk and branch breaks, and the segmentation results are less satisfactory. UNet has good overall contour segmentation under normal lighting conditions, with information missing in some details. However, prediction is poor in more complex environments, and there are significant mis-segmentation cases, such as at night and in snowy conditions. In addition, UNet incorrectly divides the background into the trunk and branches of *Abies beshanzuensis* (Figure 10c,d). The improved UNet designed in this paper, which used the SE and RAM modules, allowed the model to pay more attention to the detailed segmentation of the trunk and branching parts of *Abies beshanzuensis*, which alleviated the occurrence of under-segmentation and mis-segmentation problems under complex environmental conditions, thus resulting in a more detailed segmentation structure.

To evaluate the impact of the various components of the proposed method on the model performance, ablation experiments were designed for the *Abies beshanzuensis* dataset. UNet was chosen as the basic network structure to evaluate the impact of the VGG16, SE module, and RAM on the model performance, and the results are shown in Table 2.

Compared with the original UNet model, the VGG16 used as the backbone network deepened the layers of the network and could fit the features better. After replacing the backbone network, the MIoU and F1-scores increased by 0.4685% and 0.2657%, respectively. Adding the RAM and the SE module to the UNet network of the replacement backbone suppressed the feature weights, such as the target-independent noise, and enhanced the useful feature weights. The MIoU increased by 0.5613% and 0.6675%, respectively, and the F1-scores increased by 0.3150% and 0.3755%, respectively, demonstrating the effectiveness of the added attention mechanism. Finally, by applying both SE and RAM to the network, the MIoU and F1-scores were further improved by 1.772% and 0.986%, respectively, as compared to the replacement backbone, UNet. In summary, the use of a deeper backbone network and the embedding of the SE modules and RAM are effective, as can be inferred from the characteristics of the morphology and environment of *Abies beshanzuensis*.

To validate the performance of our improved model in different environments, we further segmented the original dataset, and the segmentation results are shown in Table 3. As seen therein, environmental conditions slightly impact segmentation performance, with sunny days having the best metrics and snowy days the worst. We believe sunny days usually provide ample natural light, making the edges and textures of trees more visible and the differences between trees and their surroundings more apparent. Such good lighting conditions help the model capture the boundaries and details of the trees accurately, thus improving the segmentation accuracy. In contrast, under snowy weather conditions, the snow cover blurred the outline and texture of the *Abies beshanzuensis*, causing some alteration in its appearance and making it more difficult to distinguish between the trees and the background, thus reducing the segmentation accuracy. However, the environmental conditions had limited influence on the segmentation, and the improved model could meet the segmentation requirements for different weather.

### 3.3. Skeleton Extraction with Length and Inclination Calculation

**Figure 11:**
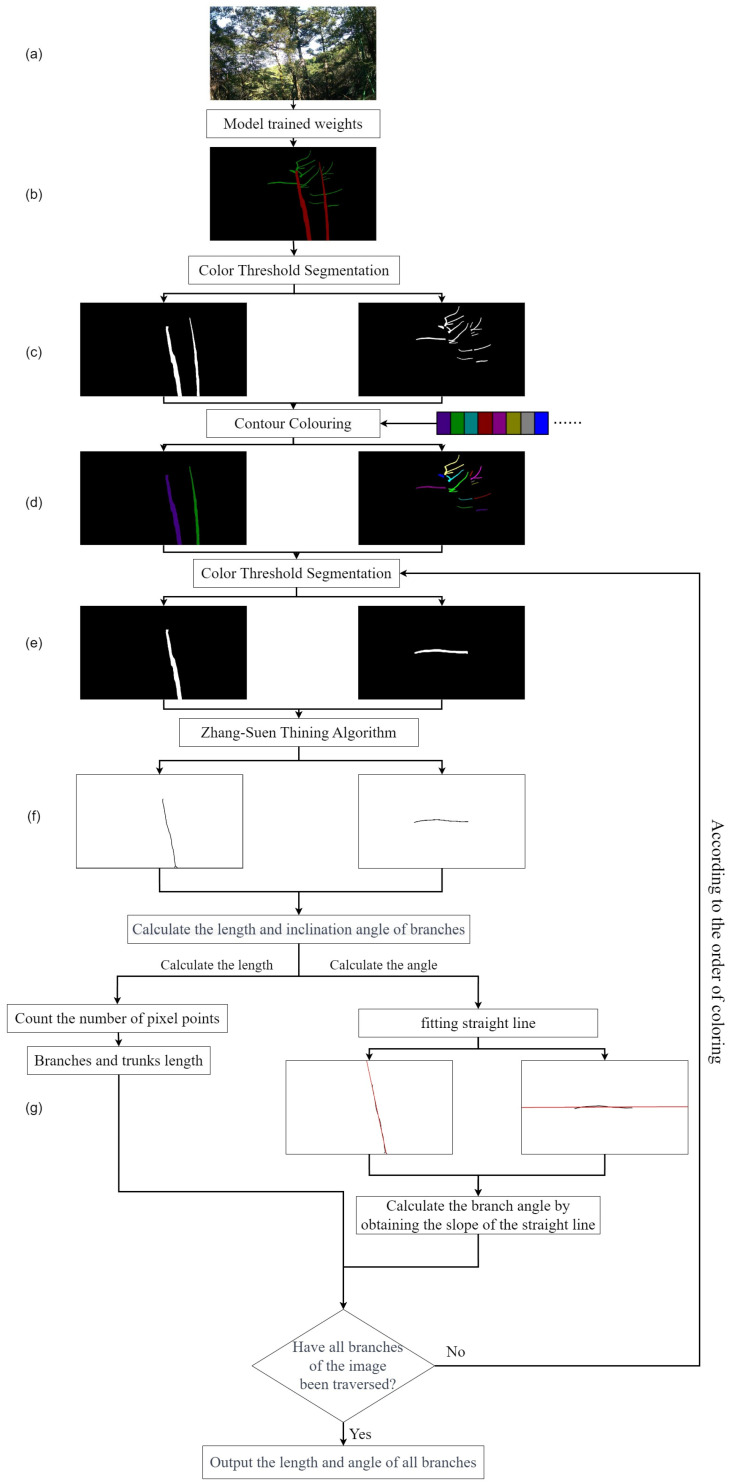
The whole process of calculating the length and inclination angle of *Abies beshanzuensis* branches. (**a**) Images captured by the camera; (**b**) Model segmentation results; (**c**) binary image of branches or trunks of the *Abies beshanzuensis*; (**d**) Fill in the color in the binary image. (**e**) Binary image of a single branch or trunk; (**f**) Single branch or trunk to obtain skeleton by Zhang&Suen thinning algorithm; (**g**) The skeleton line is fitted to a straight line through the Euclidean distance.

The flowchart in Figure 11 illustrates the method used for measuring the trunk and branch lengths, as well as the inclination angles of *Abies beshanzuensis*, which was based on the Zhang–Suen skeleton thinning algorithm. The optimized UNet model was used to segment and extract the trunk and branches to obtain the mask of the predicted image (Figure 11b). Then, the mask images of the trunk and branches were obtained via the color thresholding process (Figure 11c). Due to the limitations of the two-dimensional image, we took the overlapping branching mask as a whole. The number of trunks and branches were counted separately in the mask image, where they were marked for the first time. In order to count the branches correctly, the model segmentation error was reduced by eliminating the branches that were smaller than a certain contour area as they were considered incorrectly segmented. After that, the remaining contours in the mask were marked again, and the contours of the main trunk and branches were filled with color and used to distinguish them from each other (Figure 11d). The color-filled image was again subjected to color threshold segmentation in order to locate a single target (Figure 11e). Then, the target image was processed using the Zhang–Suen thinning algorithm, the skeleton image was extracted (Figure 11f), and the number of white pixels in the binary skeleton image was counted and considered as the length of the branch. Finally, the obtained skeleton image was fitted with a straight line using Euclidean distance to obtain the slope and determine the inclination angle (Figure 11g). The slope of this line is obtained by taking any two points on the fitted line to find the equation of the inclination of the line. For the purpose of monitoring the angle of the branch, the inclination of the branch is finally calculated using the inverse tangent function and the radian conversion factor.

### 3.4. Evaluation of Trunk and Branch Lengths and Inclination Measurement

**Figure 12:**
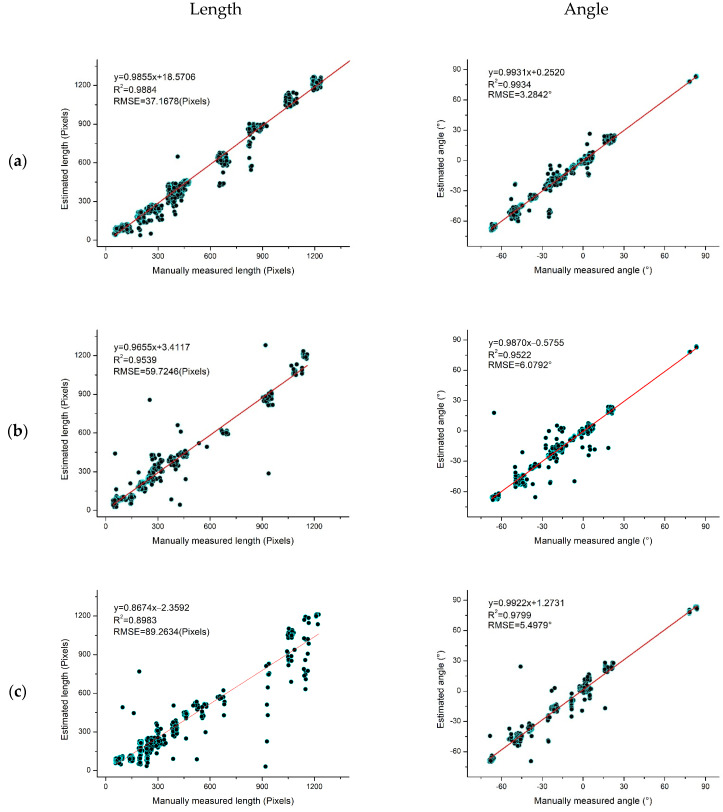

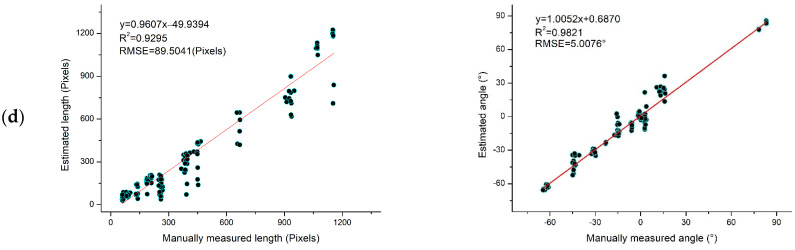
Regression analysis of manual measurements on estimated branch length (**left**) and inclination angle (**right**). (**a**) Sunny environment. (**b**) Rainy day environment. (**c**) Natural light, well-lit environment at night. (**d**) Snowy environment. Red lines are regression lines.

We measured all branches manually to evaluate the accuracy of the proposed method for branch length and inclination angle. Considering the actual situation and the complexity of field measurements, we used manually labeled images as the actual values in this paper. In addition, data on 2760 branches of *Abies beshanzuensis* were collected from August 2022 to January 2023, including 1410 branches on sunny days, 636 on rainy days, 554 branches at night with good natural light conditions, and 160 on snowy days. The comparison of the estimation results from the method proposed in this paper with the manual measurement data is shown in Figure 12. The branch length is shown on the left, and the branch inclination angle is on the right.

Figure 12a shows the correlation analysis of the branches’ actual length and inclination angle in a sunny environment along with the algorithm’s predicted length and inclination angle. By building a scatter plot and performing a correlation evaluation, the R^2^ value of both the length and the inclination angle was determined to be greater than 0.98. Figure 12b shows the correlation analysis of the actual and predicted lengths and inclination angles of 636 selected samples in a rainy environment. The corresponding R^2^ values are 0.9539 and 0.9522, respectively, which are lower as compared to those of the sunny days. Figure 12c shows the actual and predicted correlation analysis of branch length and inclination for the selected samples in a well-lit natural environment at night, with an R^2^ value of 0.8983 and an RMSE of 89.2634 Pixels for length, and an R^2^ value of 0.9799 and an RMSE of 5.4979° for inclination; the inclination identified in this environment is more accurate. However, the R^2^ value for length is the lowest among all environments. Figure 12d shows the correlation analysis between the actual length and inclination angle of the branches under snowy conditions, and the predicted length and inclination angle values. The R^2^ values are 0.9295 and 0.9821, and the RMSE values are 89.5041 Pixels and 5.0076°, respectively. The above data suggest that the R^2^ value for the evaluation of branch inclination is above 0.95, while the accuracy of the length fluctuates more with the change in environment, and that the R^2^ value is less than 0.95 at night and on snowy days.

In general, the branch lengths and inclination angles estimated via the method in this paper show a strong linear relationship with the manual measurements. They can be used as an effective means of obtaining information on branch lengths and inclination angles.

## 4. Discussion

Previous research on vision-based plant phenotypes has focused on the segmentation of crops and potted plants, and researchers have conducted numerous related studies using their respective datasets and methods. Some segmentations based on machine learning techniques have achieved good model accuracy [36,37]. Among the studies on plant trait extraction, some of the researchers used chlorophyll fluorescence signals and hyperspectral images from uncrewed aerial vehicles for the measurement of maize traits [38,39]. In addition, many scholars have also applied deep learning methods to the phenotype monitoring of plants. Kolhar et al. used the DeepLabV3+ model to segment leaves of Pinus sylvestris plants to determine three phenotypes of leaf count, projected plant area and emergence time of leaves, enabling prediction and tracking of plant growth [40]. Some of them have improved the UNet model accordingly to achieve good results [41,42]. However, some general object segmentation models based on deep learning are not fully applicable to the segmentation methods for large plants in complex background and lighting environments in the wild. This is because the trunk and branches of tall trees in complex natural environments are close to the background color, and a single branch occupies fewer pixels of the whole image, thereby making segmentation difficult. In this study, we proposed an improved UNet algorithm for segmentation under complex conditions in the field by selecting *Abies beshanzuensis* as the research object since there are only three surviving plants in the world. The four evaluation indicators of the improved algorithm all reached more than 94%. The experiments proved that the accuracy of branch segmentation can be guaranteed in various environments in the field, laying the foundation for the later branch phenotype measurement. Since this paper used semantic segmentation rather than instance segmentation, it was not possible to distinguish different instances based on specific categories. Therefore, a series of image processing methods were needed to label the branches in order to obtain the skeleton of the trunk and each branch and to finally calculate the length and inclination angle of the target object.

In order to verify the effectiveness of the method, 2760 randomly selected branch samples were tested in this paper, and the R^2^ values of their lengths and inclination angles were more significant than 0.89. It can be seen from the experimental images that the branches are relatively small and complex, and their colors are similar to that of the image background. In measuring the length, the highest accuracy achieved in the experiment was on sunny days, followed by rainy days and snowy days, while the worst accuracy was at night; in measuring the inclination, the highest accuracy achieved in the experiment was on sunny days, followed by snowy days and at night, while the worst accuracy was on rainy days. The reason for the variation is that more attention is given to the details of the branches when measuring the length, while in measuring the angle, attention needs to be paid only on the whole tree. In recent years, automated monitoring technologies based on the Internet of Things have rapidly developed and intelligent monitoring in greenhouses is also becoming increasingly widespread in plant applications [43,44]. As for the monitoring of precious plants, both the monitoring of the surrounding environment of plants and studies on the plants themselves have been conducted [45,46]. However, these studies generally require the deployment of equipment near the target or even on the target itself, which would easily affect the target and the surrounding ecological environment. The camera in this study is far away from the experimental object and thus has a relatively low impact.

Phenotypic data of branch length and inclination angle were obtained, and their real-time change values were monitored and analyzed to determine in real time whether the plant was undergoing branch breakage or external stress events. In contrast to human supervision, we showed that this combination of high-resolution images and deep learning could facilitate research on the dynamic monitoring of plants. In the follow-up research work, we will further obtain other phenotypic information, such as leaf color and bark damage area, monitor the change of plant traits, and establish a phenotypic index system for the health monitoring of tall and precious trees in the wild.

## 5. Conclusions

Currently, the monitoring and protection of precious tree species in the field environment are still mainly performed manually. Management and care measures are more traditional, resulting in the waste of human and material resources. To solve this problem, this paper proposed an improved UNet algorithm, which introduced the SE module and RAM to facilitate the fusion of multi-scale features and improved the accuracy of branch segmentation. Our experiments proved that compared to PSPNet, Deeplabv3+ and UNet networks, our network can effectively segment the branches of *Abies beshanzuensis* in the image; the MIoU of the segmentation reached 94.30%. In addition, through the Zhang–Suen thinning algorithm, the skeleton curves of the trunk and branches were obtained and the length of the pixel points was calculated. The inclination angles of the trunk and branches were also obtained via straight line fitting. The measurements achieved satisfactory accuracy, and the goal of obtaining phenotypic information on tall plants in a complex environment in the wild was accomplished. This paper also provides a technical route for the future development of intelligent monitoring standards for valuable tree species in the field and serves as a case study for deep learning in field plant health monitoring.

## Figures and Tables

**Table 1:** Performance comparison of six models.

Model	Dataset				
	Backbone	MIoU	Precision	Recall	F1-Score
PSPNet	MobileNet	71.0251	86.8085	76.7713	80.6257
	ResNet50	73.3881	87.7154	79.1478	82.4199
DeepLabV3+	MobileNet	82.8715	92.2064	87.8583	89.8485
	Xception	83.2533	91.4656	88.9019	90.1167
UNet	original	92.0595	95.8051	95.7121	95.7582
Our model	VGG16	94.2998	96.9498	97.0711	97.0103

**Table 2:** Comparison results for model performance of each module under the ablation experiments.

Model	Dataset			
	MIoU	Precision	Recall	F1-Score
UNet	92.0595	95.8051	95.7121	95.7582
UNet+VGG16	92.5280	96.0674	95.9818	96.0239
UNet+VGG16+RAM	93.0893	96.3936	96.2860	96.3389
UNet+VGG16+SE	93.1955	96.3509	96.4493	96.3994
UNet+VGG16+SE+RAM	94.2998	96.9498	97.0711	97.0103

**Table 3:** Improved model performance in different weather conditions.

Environment	Our Model			
	MIoU	Precision	Recall	F1-Score
Sunny	95.4216	97.6699	97.5869	97.6283
Rainy	93.8677	96.7305	96.8074	96.7688
Night	92.5565	95.9979	96.0106	96.0042
Snowy	92.2895	95.5902	96.0873	95.8372

## Data Availability

The image dataset used to support the findings of this study is available from the corresponding authors upon request.
